# Optimization of 3D Passive Acoustic Mapping Image Metrics: Impact of Sensor Geometry and Beamforming Approach

**DOI:** 10.3390/s24061868

**Published:** 2024-03-14

**Authors:** Sarah Therre, Marc Fournelle, Steffen Tretbar

**Affiliations:** 1 Department Ultrasound, Fraunhofer Institute for Biomedical Engineering (IBMT), 66280 Sulzbach, Germany; marc.fournelle@ibmt.fraunhofer.de (M.F.); steffen.tretbar@ibmt.fraunhofer.de (S.T.); 2 Department of Molecular and Cellular Biotechnology, Saarland University, 66123 Saarbruecken, Germany

**Keywords:** passive acoustic mapping, cavitation monitoring, high-intensity focused ultrasound, ultrasound therapy

## Abstract

Three-dimensional passive acoustic mapping (PAM) with matrix arrays typically suffers from high demands on the receiving electronics and high computational load. In our study, we investigated, both numerically and experimentally, the influence of matrix array aperture size, element count, and beamforming approaches on defined image metrics. With a numerical Vokurka model, matrix array acquisitions of cavitation signals were simulated. In the experimental part, two 32 × 32 matrix arrays with different pitches and aperture sizes were used. After being reconstructed into 3D cavitation maps, defined metrics were calculated for a quantitative comparison of experimental and numerical data. The numerical results showed that the enlargement of the aperture from 5 to 40 mm resulted in an improvement of the full width at half maximum (FWHM) by factors of 6 and 13 (in lateral and axial dimension, respectively). A larger array sparsity influenced the point spread function (PSF) only slightly, while the grating lobe level (GLL) remained more than 12 dB below the main lobe. These results were successfully experimentally confirmed. To further reduce the GLL caused by array sparsity, we adapted a non-linear filter from optoacoustics for use in PAM. In combination with the delay, multiply, sum, and integrate (DMSAI) algorithm, the GLL was decreased by 20 dB for 64-element reconstructions, resulting in levels that were identical to the fully populated matrix reconstruction levels.

## 1. Introduction

With the emergence of therapeutic ultrasound as a treatment method in various clinical scenarios, the need for adequate and reliable monitoring methods is persistently growing. MR-based approaches allow for a precise monitoring of thermal ablation and are currently the clinical standard for ultrasound therapy monitoring [1]. However, their high initial and operational costs, as well as the restricted availability in clinical environments hinder the more widespread use of ultrasound in a therapeutic context. Furthermore, when it comes to non-thermal mechanisms, the MR-based monitoring methods are of limited use only [1]. The visualization of the outcome of mechanical-induced phenomena, like histotripsy lesions or the blood–brain-barrier opening, is possible with MR methods [2]. Nevertheless, these are incapable of detecting underlying effects, such as cavitation events [1]. Ultrasound-based monitoring approaches are able to overcome these limitations, which is why extended research has been ongoing in this direction in recent years [3,4,5,6,7,8]. 

Besides heating, cavitation may occur during the application of therapeutic ultrasound. Whether this is desired in the specific context or not, a reliable monitoring of the treatment site concerning the appearance of cavitating bubbles is crucial. In particular, in scenarios not concerned with tissue ablation, such as transient blood–brain barrier opening or neurostimulation [9,10,11], the occurrence of inertial cavitation signals suggests prohibitively high pressure levels, requiring an immediate cessation of sonication in order to avoid undesired non-reversible damage, which highlights the need for a reliable monitoring approach. The oscillating and potentially collapsing bubbles act as secondary acoustic sources [12]. Their characteristic emissions can be recorded using a separate ultrasound array in receive mode, thus allowing the spatial and temporal reconstruction of the cavitation sources. These methods are generally referred to as passive acoustic mapping (PAM), as the monitoring transducers do not actively send ultrasound signals and can therefore be considered passive. Until now, numerous reconstruction algorithms in the time domain and in the frequency domain have been proposed [6,13,14,15,16,17,18]. Additionally, a distinction can be made between data-adaptive and non-adaptive algorithms.

The most common algorithm for PAM, known under the name of time exposure acoustics (TEA), is based on the conventional delay-and-sum beamforming and was first introduced by Salgoankar et al. [13]. Data-adaptive algorithms (e.g., Robust Capon Beamforming) can improve the rather poor image quality of non-adaptive algorithms like TEA, as the signals are weighted individually to suppress interference artefacts [15]. These data-adaptive algorithms typically suffer from a high computational complexity [19,20]. To improve the axial resolution while maintaining a low computational complexity, Bae et al. introduced a coherence factor as a weighting parameter in combination with TEA [20]. Another algorithm with rather low computational costs is the delay, multiply, sum, and integrate algorithm (DMSAI), introduced by Lu et al. for PAM [16]. Despite its simplicity, the algorithm is able to produce superior image quality when compared to other non-adaptive algorithms [16]. A method with very high computational speed is the angular spectrum method (ASM), which operates in the frequency domain and was adapted for PAM by Arvanitis et al. [14]. Although the algorithm is only able to obtain a rather poor image quality that is strongly dependent on the transducer’s element pitch, the comparably fast reconstruction offers great potential regarding real-time monitoring. In this study, as the point spread function (PSF) size for different array configurations was of major interest, we chose DMSAI for 3D-PAM. 

Linear arrays, which are commonly chosen for PAM, allow the reconstruction of single planes in a certain orientation to the therapeutic transducer. Therefore, the adequate alignment of the linear array is crucial for the outcome of the reconstruction, since out-of-plane cavitation events will lead to artefacts that suggest cavitation at erroneous locations. The use of matrix arrays offers the possibility of monitoring cavitation not only in a plane, but also in a whole volume surrounding the therapeutic transducer’s focus area. Thus, the 3D reconstruction of cavitation events could make treatment monitoring potentially even safer due to the chance of missing cavitation events being diminished. However, the large number of channels typically employed in systems capable of driving matrix arrays poses significant demands on the receiving electronics and leads to a high computational load, along with challenges regarding memory allocation and data transfer. Therefore, it would be favorable to use fewer elements for the reconstruction of 3D cavitation maps. The aperture size of matrix arrays differs depending on the array’s purpose. For passive beamforming algorithms with linear arrays, the aperture size has already been identified as a key parameter for the outcome of the reconstruction, with large apertures being favorable [16,21]. Recently, Schoen et al. were able to draw similar assumptions for ASM reconstructions with simulated matrix arrays [22]. Furthermore, Sivadon et al. numerically investigated random and regular sparse arrays for use in PAM, finding that a reduction from 1024 to 256 elements resulted in nearly identical images [23]. This is consistent with a simulation study from Acconcia et al., who suggested the modest number of 128 receiver elements to be sufficient for PAM [24]. Liu et al. also found different levels of sparsity to be suitable for receiving array configurations [25]. Jones at al. performed extensive simulation studies concerning the number of receivers in hemispherical arrays for 3D-PAM with TEA and translated their findings to an experimental evaluation of a hemispherical array with up to 256 sparsely distributed receivers [26,27]. Nevertheless, to our knowledge, no closer evaluation regarding the influence of the aperture sizes and the number of elements of matrix arrays has been conducted as of yet, especially with both numerical and experimental data for as many as 1024 receiving elements on a diagnostic imaging array and with DMSAI as the reconstruction algorithm.

This study aims to close this gap by investigating the influence of the number of elements and aperture size of a matrix array on the reconstruction quality, both numerically and experimentally. We furthermore compare DMSAI with TEA for reconstructions with 64 elements and introduce a standard deviation based (STD)-filter to suppress grating lobes and further improve the PSF size for both algorithms [28].

## 2. Materials and Methods

### 2.1. Simulation Model

A numerical model was implemented to simulate the acquisition of cavitation signals by ultrasound arrays. The so-called multi-bubble-model was introduced by Vokurka and was adapted for PAM by Coviello et al. [15,29]. By applying individual delays to the cavitation signals generated by *K* bubbles, the recorded signal *p* of an array element *I* can be written as follows:

(1) $$p_{i}\left(t\right)=\sum_{n=-1000}^{1000}\sum_{k=1}^{K}\frac{p_{kn}}{d_{i}\left(x_{k}\right)}\exp\left(\frac{-\left|t-nT_{0}-\phi_{kn}-\tau_{i,k}\right|}{\theta_{kn}}\right)$$

The pulse period *T*_0_ is defined by the excitation frequency *f*_0_. *ϕ_kn_* is the phase offset of one pulse period, *θ_kn_* is the time constant, and *p_kn_* is the maximum acoustic pressure. All three parameters are assumed to be random variables that are normally distributed. The values for the random distribution were chosen to be the same as used by Coviello et al. [15]. In Table 1, the values for the different parameters are listed.

The division by the distance *d_i_(**x**_k_)* from each cavitation bubble *k* at the position ***x****_k_* = [*x_k_,y_k_,z_k_*]*^T^* to the different array elements *i* accounts for the spherical spreading of the pressure wave. Combined with the spatially constant speed of sound *c*, the time of flight from a cavitation bubble to an array element can be calculated via *τ_i,k_* = *d_i_(**x**_k_)/c*.

In this study, a single source consisting of two bubbles (in a water environment) was simulated at different positions (centered or off-axis, see Table 2). The simulated cavitation sources at (0/0/50) mm and (0.9/−5.3/50) mm were chosen, as these correspond to the cavitation positions extracted from the experimental data. Additionally, one cavitation source in a different depth ((0/0/40) mm) and one off-axis cavitation source ((6/−5/50) mm) were both simulated for the evaluation of the sparsity schemes.

**Figure 1:**
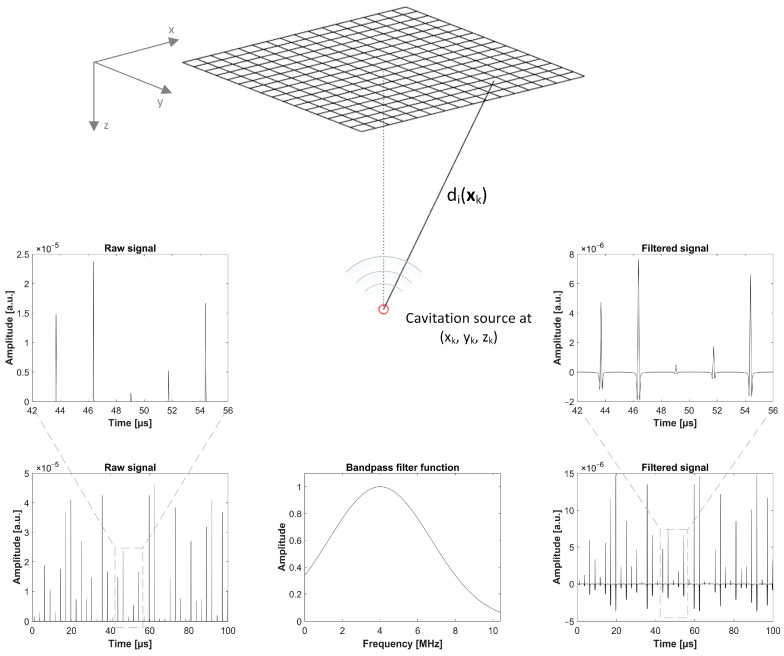
Visualization of the numerical model. Simulation setup and examples of a simulated raw signal received by one channel, the applied transfer function, and the resulting filtered signal. The numerical model is based on the multi-bubble model of Vokurka, as adapted for passive acoustic mapping by Coviello et al. [15,29].

The simulated acquisition parameters were chosen to match the experimental characteristics. Accordingly, the excitation frequency was set to 375 kHz and the diagnostic transducer was assumed to acquire signals for 90 µs, with collapsing bubbles being present throughout the whole time period. In order to mimic the transducer’s bandwidth, a spectral bandpass filter (simulated Gaussian transfer function) was applied to the simulated signal. The center frequency and bandwidth of the bandpass filter were chosen according to the simulated transducer configuration (4 MHz with 80% −6 dB bandwidth and 2.84 MHz with 85% −6 dB bandwidth, respectively). The simulated setup, as well as examples of a raw signal received by one channel, the applied transfer function, and the resulting filtered signal are all shown in Figure 1. To account for the element directivity of the receiving transducers, each element was divided into 121 subelements. This corresponds to the subelement sizes of 80 µm and 20 µm, respectively, being significantly smaller than the considered wavelengths. The cavitation signals were generated for each subelement and the final cavitation signal of one element was created via the summation of all 121 subelement signals.

**Figure 2:**
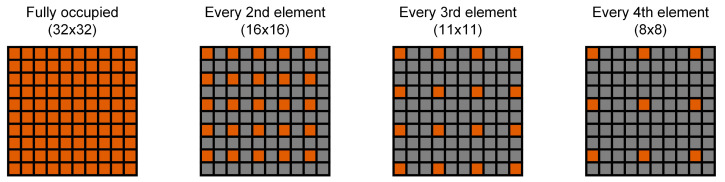
Visualization of sparse array generation. Schematic drawing of the principle of thinning out a fully populated matrix, resulting in sparse arrays with equally spaced elements.

To assess the influence of the number of elements and the array size of the matrix arrays on the reconstruction quality, different monitoring transducer configurations were simulated. In the first step, the number of elements was varied. The chosen values for the number of elements and the corresponding pitch values can be seen in Table 3. For these setups, a center frequency of 4 MHz and a −6 dB bandwidth of 80% was assumed, paired with an element size of 0.88 × 0.88 mm^2^. Four different positions of the cavitation source were simulated for the evaluation of the sparsity schemes, two of which were also available in the experimental data (see Table 2). The simulated array configurations were chosen to ensure that they can be experimentally realized through the use of the subsets of the existing 32 × 32 matrix with a pitch of 0.94 mm, taking every second, third, or fourth element in each row and column. This leads to slightly asymmetric configurations for the 16 × 16, 11 × 11, and 8 × 8 setups, and, therefore, to slightly decreased aperture sizes (see Figure 2). For the 63 × 63 setup, the border elements were defined to be at the same position as for the 32 × 32 variant, which also results in a slightly decreased aperture size. In the second step, a variation of the aperture sizes was simulated, while the number of elements stayed constant at 32 × 32. Table 4 shows the simulated different aperture sizes, where the number of elements was set to 32 × 32 for all setups. Here, the acquisition of a cavitation source at (0/0/50) mm was simulated for all aperture configurations, one time with an element size of 0.88 × 0.88 mm^2^, a center frequency of 4 MHz, and an 80% −6 dB bandwidth for all configurations, and one time with an element size of 0.24 × 0.24 mm^2^, a center frequency of 2.84 MHz, and a −6 dB bandwidth of 85%. In both tables, the bold numbers represent the values that were available in the experimental setup.

### 2.2. Reconstruction Algorithms

#### 2.2.1. Delay, Multiply, Sum, and Integrate

The delay, multiply, sum, and integrate (DMSAI) algorithm was implemented following the example set by Lu et al. [16]. A brief description of the algorithm is provided hereafter.

After defining a voxel grid in the region of interest, the recorded signals *p* of each sensor element *i* are delayed individually, based on the distance to each voxel. The delayed signal *s_i_* can be written as

(2) $$s_{i}\left(x,t\right)=d_{i}\left(x\right){\;p}_{i}\left(t+\frac{d_{i}\left(x\right)}{c}\right),$$

where *d_i_(**x**)* accounts for the spherical spreading. In the DMSAI algorithm, the delayed signals are then combinatory coupled, multiplied, and summed over the number of channels *N* to obtain the source strength *q* as follows:

(3) $$q\left(x,t\right)=\sum_{i=1}^{N-1}\sum_{j=i+1}^{N}s_{i}\left(x,t\right)s_{j}\left(x,t\right).$$

After some simplification steps that are not shown, the source strength results in

(4) $$q\left(x,t\right)=\left[S_{1}^{2}\left(x,t\right)-S_{2}\left(x,t\right)\right]/2,$$

where $S_{1}\left(x,t\right)=\sum_{i=1}^{N}{\bar{s}}_{i}(x,t)$, $S_{2}\left(x,t\right)=\sum_{i=1}^{N}{\bar{s}}_{i}^{2}(x,t)$, and ${\bar{s}}_{i}\left(x,t\right)=\mathrm{sign}\left[s_{i}\left(x,t\right)\right]\sqrt{\left|s_{i}\left(x,t\right)\right|}$. The source strength is then squared and summed over a certain time period *T*, resulting in the source intensity *I*, which defines the amplitude of the cavitation map for each voxel.

(5) $$I\left(x\right)=\frac{4\pi }{N^{2}\rho c}\int_{t_{0}}^{t_{0}+T}q{\left(x,t\right)}^{2}dt$$

#### 2.2.2. Time Exposure Acoustics

For the time exposure acoustics algorithm, which is another approach typically used in time-domain PAM beamforming, the source strength *q* is defined as [7]

(6) $$q\left(x,t\right)=\sum_{i=1}^{N}s_{i}\left(x,t\right).$$

The source intensity *I* for each voxel is then obtained by substituting Equation (6) into Equation (5).

#### 2.2.3. Standard Deviation-Based Filter

Analogous to the original implementation of the standard deviation (STD)-based filter for the use in optoacoustic imaging, we implement the inverse standard deviation of the delayed signals *s_i_* as a weighting factor in PAM [28]. Neglecting the differences in element sensitivity (both manufacturing related and due to different incidence angles), the amplitude of the delayed signal at time *t* and voxel position ***x*** should be constant for all elements, if the considered voxel corresponds to the position of a cavitation source. The deviation from this pattern, defined as the inverse relative standard deviation of the delayed signals, can then be used as the weighting factor *σ* for each voxel at the time sample *t* with

(7) $$\sigma \left(x,t\right)=\frac{\frac{1}{N}\sum_{i=1}^{N}s_{i}(x,t)}{\sqrt{\begin{array}{c} \frac{1}{N}\sum_{i=1}^{N}{\left(s_{i}\left(x,t\right)-\frac{1}{N}\sum_{i=1}^{N}s_{i}\left(x,t\right)\right)}^{2} \end{array}}}$$

By multiplying *σ* voxel per voxel with the PAM reconstruction for each time sample *t*, the factor acts as an image filter. This STD filter is applied for both DMSAI and TEA-reconstructions, thus altering Equation (4) for DMSAI to

(8) $$q\left(x,t\right)=\left[{\left(\sigma \left(x,t\right)S_{1}\left(x,t\right)\right)}^{2}-{\sigma \left(x,t\right)S}_{2}\left(x,t\right)\right]/2,$$

and Equation (6) for TEA to

(9) $$q\left(x,t\right)=\sigma (x,t)\sum_{i=1}^{N}s_{i}\left(x,t\right).$$

### 2.3. Performance Metrics

In our study, data from transducers with different aperture sizes and different numbers of elements were reconstructed with DMSAI. The performance of the different transducer setups was evaluated using the FWHM values in lateral and axial directions, as well as the background contrast. These three parameters were chosen as primary metrics because the main interest of the study was to assess the PSF size of cavitation bubbles (reflected by FWHM values), as well as the ability of the reconstruction chain to suppress artifacts (reflected by the background contrast). All metrics were calculated from the reconstructed RF-Data (Intensity *I* from Equation (5)), without any compression or scan conversion being applied. In the numerical part, the localization error with respect to the position of the simulated source was calculated as the distance between the known source location and the reconstructed maximum. Initially, a defined volume in the region of interest was reconstructed. The step size was set to 0.3 mm in the x/y-direction and to 0.5 mm in the z-direction. The background contrast or signal-to-noise ratio (SNR) of the reconstructed volume was then assessed according to the definition of Lu et al. [16].

(10) $$SNR=10\log_{10}\left(\frac{I_{higher}}{I_{lower}}\right)$$

*I_higher_* is the mean of the intensity values above half of the maximum intensity and *I_lower_* is the mean of the intensity values that are below or equal to half of the maximum intensity. In order to calculate the FWHM, the location of the maximum intensity in the reconstructed volume was determined. In the lateral direction, a single line with a step size of 0.05 mm was reconstructed at the elevational and axial coordinate of the maximum intensity. Analogous to that, a single line in the axial direction was reconstructed at the elevational and lateral maximum, this time with a step size of 0.1 mm. For selected setups, the volume surrounding the detected maximum was reconstructed with a step size of 0.1 mm in all directions for imaging purposes. To assess the influence of the element count on the reconstruction time, the mean value of five reconstructions was taken using the MATLAB stopwatch function on a Dell Inc. (Round Rock, TX, USA) Precision 5820 Tower (Intel^®^ Xeon^®^ W-2235, CPU @ 3.8 GHz, 32 GB RAM) for a reconstruction volume of 33 × 33 × 64 voxel.

### 2.4. Experimental Setup

**Figure 3:**
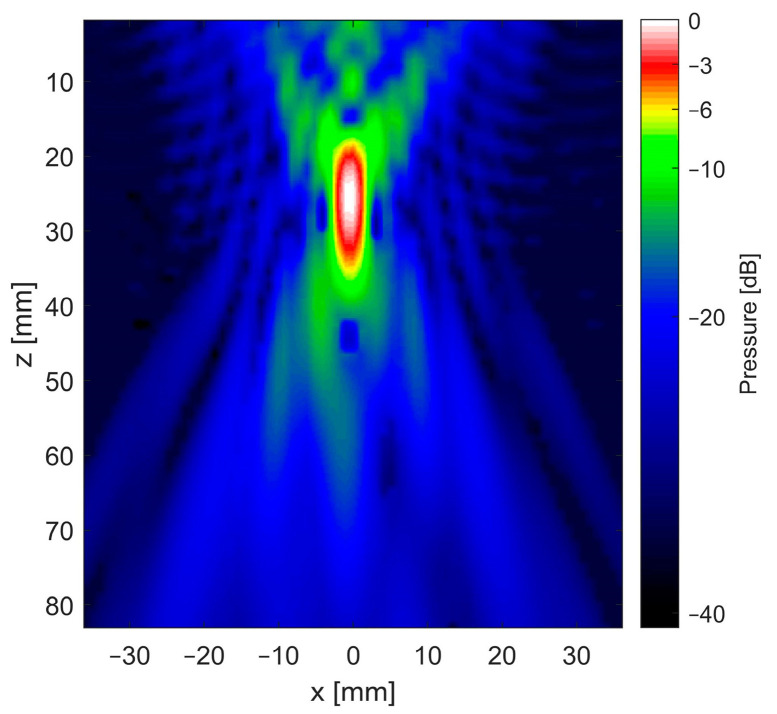
Sound field of 375 kHz focused transducer used for the generation of cavitation bubbles.

A custom-made spherical focusing transducer (Fraunhofer IBMT, St. Ingbert, Germany) with a frequency of 375 kHz was used to generate cavitation bubbles in a small plastic tank filled with water at room temperature. This well-characterized in-house developed transducer was chosen because it had been used previously to successfully generate cavitation bubbles in similar setups. The X/Z plane pressure distribution field of the transducer, which was acquired by scanning a calibrated hydrophone (Type s, RP acoustics e.K., Leutenbach, Germany) in steps of (Δx = Δz) = (1 mm) in front of the aperture using a programmed motion stage, can be seen in Figure 3. Combined with a power amplifier with an integrated signal generator (AG series amplifier, T&C Power Conversion Inc., Rochester, NY, USA), a continuous wave (CW) signal was generated at an electric power level of 3 W (corresponding to a peak negative pressure of approximately 1.1 MPa), as this was found to be sufficient to produce cavitation bubbles in the focus area.

Two different 32 × 32 matrix arrays were used as receivers. One was a 3 MHz 32 × 32 array (Vermon, Tours, France) with an element pitch of 0.3 mm in both directions and the other one was a custom-made 4 MHz 32 × 32 array (Fraunhofer IBMT) with a pitch of 0.94 mm. Both transducers were aligned perpendicularly to the acoustic axis of the therapeutic transducer at an axial distance of approximately 50 mm, positioned to capture the focus area of the therapeutic transducer. 

**Figure 4:**
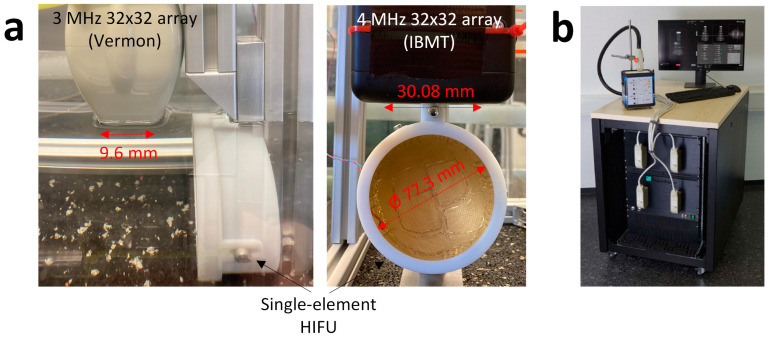
Experimental setup for the acquisition of cavitation signals in a water tank. (**a**) Side view of the 3 MHz 32 × 32 array and the single-element focusing transducer, as well as the front view of the 4 MHz 32 × 32 transducer and the single-element focusing transducer. (**b**) 1024 channel ultrasound research platform.

The pre-beamformed channel data were recorded for 90 µs after the start of sonication with the 375 kHz transducer at a sampling rate of 40 MSa/s with a 1024 channel-ultrasound research platform (Digital Phased Array System, Fraunhofer IBMT [30]), later being transferred to a separate computer for further processing with MATLAB (R2022a, The MathWorks Inc., Natick, MA, USA). A constant sound speed of 1480 m/s was assumed for reconstruction. The experimental setups with both transducers and the receiving electronics are shown in Figure 4.

## 3. Results

**Figure 5:**
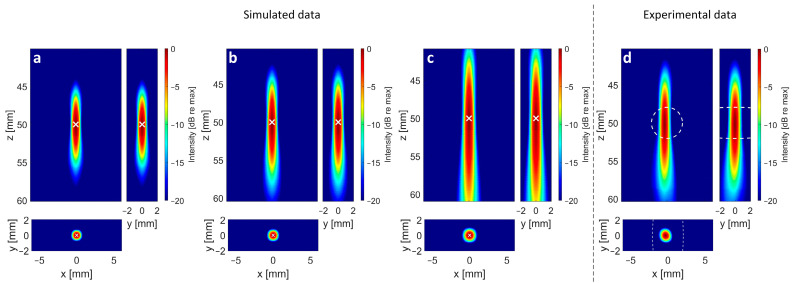
Delay, multiply, sum, and integrate (DMSAI) reconstructions for transducer setups with different aperture sizes. (**a**) Cross sections from reconstruction with numerical data, 32 × 32 elements on simulated 40 mm aperture. (**b**) Cross sections from reconstruction with numerical data, 32 × 32 elements on simulated 30.08 mm aperture. (**c**) Cross sections from reconstruction with numerical data, 32 × 32 elements on simulated 20 mm aperture. In (**a**–**c**), X shows the position of the simulated cavitation source at (*x/y/z*) (0/0/50) mm. (**d**) Cross sections from reconstruction with experimental data, 32 × 32 elements on experimentally available 30.08 mm aperture. Dashed line shows −6 dB contour of the 375 kHz transducer pressure distribution field. Images are displayed with a dynamic range of 20 dB.

In the experimental part of the study, two 32 × 32 matrix arrays with different aperture sizes as described above were used to acquire cavitation signals. The reconstruction yields 2D cross sections in X/Y, X/Z, and Y/Z directions, such as can be seen in Figure 5. Furthermore, 1D-PSFs were subsequently extracted and analyzed with respect to the FWHM for the different configurations. This process was repeated for the different simulated array configurations and the available experimental configurations described in Table 3 and Table 4.

### 3.1. Impact of Aperture Size

In Table 5 and Table 6, the lateral and axial FWHM, the SNR, and the localization errors are shown for the different aperture sizes of the transducers used in the numerical and the experimental sections. For Table 5, the generated signals correspond to receive elements with a size of 0.88 × 0.88 mm², with a center frequency of 4 MHz and a bandwidth of 80%, which corresponds to the experimentally available 32 × 32 array at 4 MHz. For Table 6, element sizes of 0.24 mm combined with a center frequency of 2.84 MHz and a bandwidth of 85% were assumed for all setups, as this fits the second available 32 × 32 array with a 9.6 mm aperture. The numerical analysis shows that the lateral FWHM can be reduced by a factor of 6 and the axial FWHM can be reduced by a factor of 13 when enlarging the aperture from 5 × 5 mm² to 40 × 40 mm² for a given number of elements and a constant element size. For smaller elements, despite the lower frequency, the improvement increases to lateral and axial factors of 11 and 20, respectively (Table 6). The visible trend (larger aperture size leads to smaller FWHM) in our simulation data could be confirmed by the experiments performed with the available 32 × 32 element matrix transducers (see Figure 4a), and even the absolute values of the FWHM are in good agreement. As the number of elements and the frequency, as well as the assumed opening angle, stay constant in the numerical studies, the positive effect on the PSF size can be directly linked to the larger aperture size. As the aperture grows, the influence of the elements’ opening angle increases, and therefore mitigates the positive effect of the aperture size on the FWHM size. Furthermore, the increased aperture size seems to be beneficial in terms of localization accuracy. 

**Figure 6:**
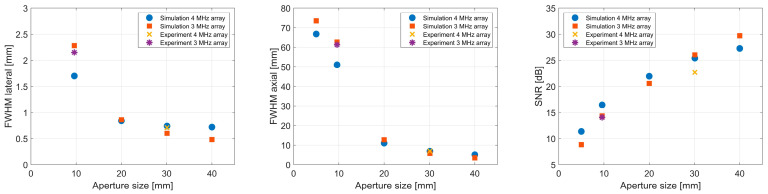
Lateral and axial FWHM values and SNR for DMSAI reconstructions with 32 × 32 elements as a function of aperture size. Numerical and experimental data for 4 MHz array correspond to values in Table 5. Data labeled as “3 MHz array” correspond to values in Table 6.

Finally, we assessed the SNR in the reconstructed data as a function of the aperture size (for constant element count). As expected, the SNR increases with a larger aperture, both in the experimental and numerical data. To illustrate the relationship between aperture size and SNR, as well as the FWHM values, the values from Table 5 and Table 6 are plotted against the corresponding aperture size (Figure 6).

### 3.2. Impact of Element Count

For both the numerical and experimental data, the FWHM and the SNR showed only minor changes when the number of elements was reduced, as shown in Table 7. The fact that the FWHM values are even smaller for a lower number of elements might be due to the slight asymmetry of the element grid, with respect to the assumed cavitation source location ((*x/y/z*) = (0/0/50 mm)), starting from the 16 × 16 array down. This asymmetry leads to slightly rotated PSFs (the PSF-axis in the z-direction always points to the center of the array), such that the FWHMs (assessed along the axes of the coordinate system) might be slightly overestimated. The localization error also ranges in a sub-millimeter range for all configurations, showing almost no degradation for lower element counts. These observed trends are consistent for both numerical and experimental data and could be confirmed for three other positions (two off-axis, one in different depth), which can be seen in Table A1, Table A2 and Table A3. The reconstruction times are shown for a relative quantitative comparison. Of course, the algorithm was neither GPU-implemented nor run-time optimized in any way, but it displays the potential of accelerating the volumetric reconstruction using a reduced element count.

**Figure 7:**
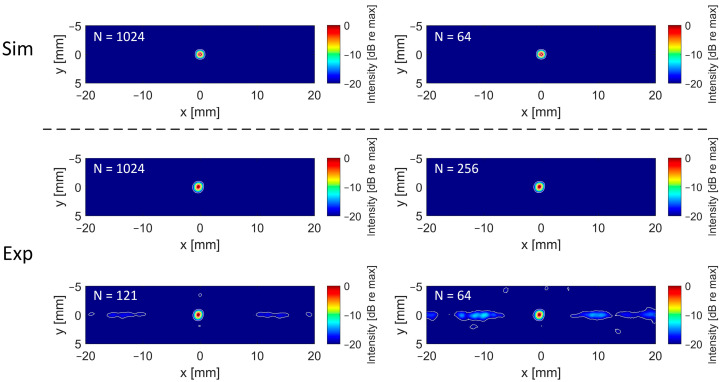
Reconstructed X/Y planes at the location of maximum intensity for the simulated data (top, cavitation source at (0/0/50) mm, received by 30.08 mm aperture) and for the experimental data (bottom, acquired with 4 MHz 32 × 32 array with 30.08 mm aperture). The number of elements on a regular grid used for each reconstruction is given by N. Images are displayed with a dynamic range of 20 dB. Contours are drawn at −3, −6 and −20 dB.

**Figure 8:**
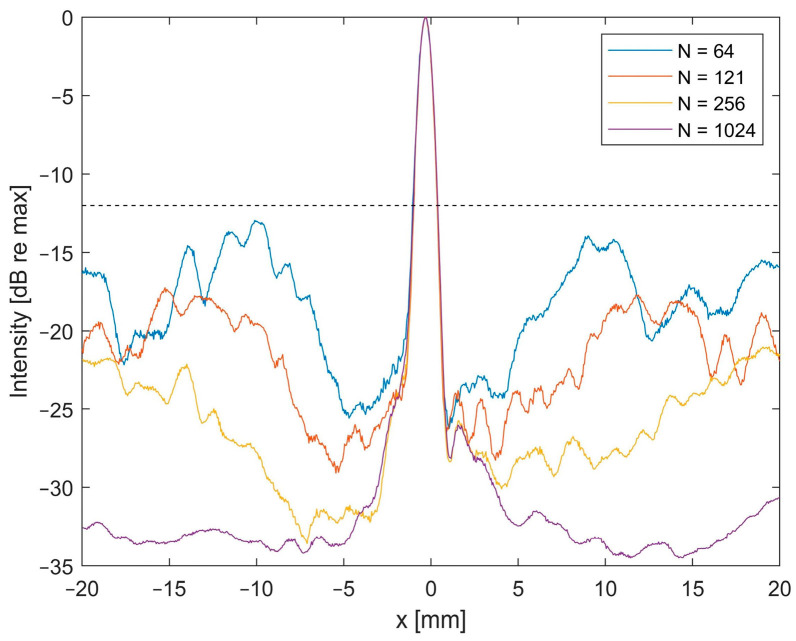
Lateral line at the elevational and axial maximum, reconstructed from experimental data with different numbers of elements (N) equally distributed on the 30.08 mm aperture. Dashed line indicates −12 dB level. Data were recorded with the 4 MHz 32 × 32 array and the data set corresponds to the experimental reconstruction shown in Figure 7.

Figure 7 shows the reconstructed X/Y planes with different numbers of elements, and a high correlation can be observed between the numerical and experimental data. As expected, when increasing the sparsity, grating lobes arise. However, even for the highest sparsity evaluated in this study (8 × 8 elements), the grating lobes remain more than 12 dB below the main lobe level for the experimental reconstruction (see Figure 8).

### 3.3. Impact of Reconstruction Algorithm and Filter for High Sparsity Schemes

**Figure 9:**
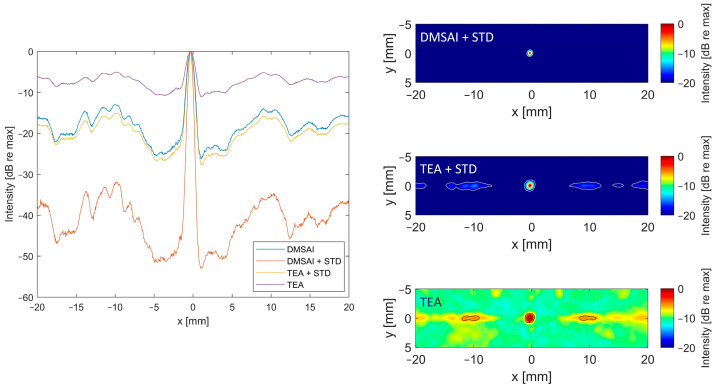
Lateral line at the elevational and axial maximum (left) and X/Y planes at the axial maximum (right), reconstructed with different reconstruction algorithms from experimental data with 8 × 8 elements equally distributed on the 30.08 mm aperture. Data were recorded with the 4 MHz 32 × 32 array and the data set corresponds to the experimental reconstruction shown in Figure 7 and Figure 8. Images are displayed with a dynamic range of 20 dB. Contours are drawn at −3, −6 and −20 dB.

As high grating lobe levels could be detrimental for the correct source localization in PAM, it is important to keep them as low as possible. Therefore, we introduce a standard deviation-based filter for use in PAM, aiming to mitigate the increased grating lobe levels caused by high sparsity levels. Figure 9 shows experimental reconstructions with the 8 × 8 element configuration for TEA, DMSAI, and their respective combination with the introduced STD filter. Both the lateral line and the X/Y plane show the poor ratio between the main lobe and grating lobe levels for TEA. In combination with the STD filter, the contrast improves, and the grating lobe level is similar to the DMSAI reconstruction. 

The combination of DMSAI and our standard deviation-based filter is able to suppress the grating lobes to the level of DMSAI reconstructions with a fully populated matrix (see Figure 8). Furthermore, the lateral FWHM improves to 0.40 mm for DMSAI plus STD, compared to 1.03 mm for TEA, 0.56 mm for TEA plus STD, and 0.71 mm for DMSAI. For the axial FWHM, which is typically poor in PAM implementations, DMSAI plus STD also yields a clear improvement (3.52 mm, compared to 10.22 mm for TEA, 5.30 mm for TEA+STD, and 6.52 mm for DMSAI).

## 4. Discussion

The goal of our study was to investigate the impact of the aperture size and the element number of a matrix array on the achievable PSF size and localization accuracy in 3D-PAM, with the ultimate goal of providing guidelines for 3D-PAM imaging systems with the lowest possible hardware requirements (e.g., in terms of channel count). Besides our results obtained from the numerically simulated data, we were able to evaluate the impact of the element count and aperture size using the experimental data acquired with our in-house developed matrix array probes, single-element focused HIFU transducers, and multichannel systems for the acquisition of the cavitation signals. 

We were able to show, with both numerical and experimental data, that the aperture size has a considerable impact on the quality of the cavitation maps reconstructed with DMSAI, while the number of elements plays a subordinate role. At a constant aperture size, a large number of elements is not favorable in terms of the achievable FWHM and SNR, i.e., the PAM reconstruction quality was almost constant for pitch values between 1.27 λ and 10.16 λ. Instead, using fewer elements on a large aperture offers the possibility of lowering the time needed for reconstruction and the requirements for the receiving electronics significantly without a degradation in PSF size and accuracy. A reduced number of elements distributed over a large aperture appears to produce images with very comparable PSFs when compared to the same aperture size with a higher element count, which is consistent with the findings of Jones et al. in their simulation study [26]. They also reported increased sidelobe levels in TEA reconstructions for a reduced number of elements, which is consistent with our findings [26]. For the DMSAI reconstructions in our study, however, only very low sidelobe levels could be detected in the experimental reconstructions, starting from 121 elements down. This might be linked to the ability of DMSAI to reduce sidelobe levels significantly, as shown by Lu et al. for 2D-reconstructions, highlighting the suitability of DMSAI for 3D-PAM [16]. Therefore, it can be suggested that the number of elements has a low impact on the PSF size and accuracy for 3D-PAM, while it is crucial that the elements are distributed over a large aperture. 

Our conclusions from Table 5 and Table 6 are consistent with the work of Schoen et al., where a large aperture seemed to have a positive influence on the reconstruction of simulated data [22]. To further explore this valuable approach, our study additionally included the variation of the pitch in order to keep the number of elements at a constant level, while increasing the aperture size, thus eliminating the possible positive influence of an increased number of elements. We were also able to go beyond the numerical evaluation through the use of two different 1024-element matrix arrays to confirm the numerical findings. With that, we could show the influence of the element count and aperture size for DMSAI reconstructions on an experimental basis with as many as 1024 elements.

For a reduced element count, the adequate choice of the reconstruction algorithm in combination with a filter allows for the effective suppression of grating lobes, as well as further improvements of PSF size. With DMSAI and the introduced STD filter, the reconstructions with 64 elements show grating lobe levels as low as in the DMSAI reconstructions with 1024 elements, emphasizing that a coarse pitch can be compensated to some extent by the proper reconstruction chain. Evidently, the addition of a filter to the signal processing chain increases the reconstruction time when compared to the original algorithm. Accordingly, a tailored solution needs to be defined for different application scenarios, depending on the chosen transducer configuration, the desired accuracy, tolerated grating/side lobe levels, and reconstruction time. 

Several limitations of this study need to be acknowledged and should be addressed in future research. First, the experiments were performed in a water environment with negligible damping characteristics, which allowed the acquisition of high harmonics, resulting from excitation with the 375 kHz therapy transducer. As the direct transferability to in vivo scenarios was not an initial aim of this study, we chose this rather basic but efficient experimental setup. However, for more realistic experiments, a tissue-mimicking phantom should be used, possibly in combination with a skull, if transcranial applications are of interest. Second, to further evaluate the performance of DMSAI in combination with the STD filter for high sparsity transducers, the simultaneous presence of multiple cavitation sources needs to be considered both in the numerical and the experimental investigations. The ability of the reconstruction chain to resolve two adjacent sources is a particularly crucial feature requiring investigation. Third, the spatial element distribution for reduced element counts has not yet been optimized. Other sparsity schemes such as random or spiral patterns should be compared to the applied regular grid.

## 5. Conclusions

In the present study, we investigated the impact of matrix array aperture size and element count on 3D-DMSAI reconstructions. We were able to show, both numerically and experimentally, that a large aperture size is crucial for improving FWHM and localization accuracy in reconstructed 3D-PAM images. Simultaneously, we could show that a fully populated matrix is not necessary for an ideal reconstruction. Even with a pitch reduced from 1.3 λ to 10.2 λ (for constant aperture size), only a minor impact was observed on the PSF, SNR, and sidelobe level. The localization accuracy also remained nearly unchanged for all tested element counts, which confirms the suitability of DMSAI for high-quality reconstructions with spatially sparse data, opening the window for 3D-PAM being used with low hardware requirements and, consequently, at a low cost. The reconstruction time was decreased by a factor of 19 when using 8 × 8 instead of 32 × 32 elements, demonstrating the potential of accelerating 3D reconstructions via the use of a reduced element count. Because the element count has a direct impact on the demands of the digitization electronics, further research will aim towards the optimization of PAM reconstruction performances under the constraint of a low element count. In particular, irregular (e.g., sparse/random matrix) geometries, already having proven their potential for volumetric imaging when using a comparably low element count, will be investigated for their suitability in PAM [31,32]. Furthermore, the implementation of 3D-PAM for transcranial applications of therapeutic ultrasound is aimed for. Phase aberrations caused by the skull need to be compensated for in the receiving part in order to correctly localize cavitation bubbles, as shown, for example, by Jones at al. [26].

## Figures and Tables

**Table 1:** Chosen parameters for the numerical cavitation model.

Parameter	Value
Phase offset *ϕ_kn_*	1 µs ± 14 ns
Time constant *θ_kn_*	2 ns ± 0.5 ns
Peak acoustic pressure *p_kn_*	3 MPa ± 10 kPa
Speed of sound *c*	1480 m/s

**Table 2:** Simulated 3D positions of the cavitation source for the evaluation of receiving sparsity schemes and aperture sizes, respectively.

Positions (*x/y/z*) for Sparsity Schemes [mm]	Positions (*x/y/z*) for Aperture Sizes [mm]
**(0/0/50)**	**(0/0/50)**
(0/0/40)	-
**(0.9/−5.3/50)**	-
(6/−5/50)	-

Bold coordinates show positions for which experimental data are available.

**Table 3:** Different numbers of elements used in the numerical studies and the corresponding element pitch and aperture size. The 32 × 32 variant with the 0.94 mm pitch is taken as the basis aperture size for all other configurations.

Aperture Size [mm]	Element Pitch [mm]	Number of Elements
29.61	0.47	63 × 63
**30.08**	**0.94**	**32 × 32**
**29.14**	**1.88**	**16 × 16**
**29.14**	**2.82**	**11 × 11**
**27.26**	**3.76**	**8 × 8**

Bold numbers show experimentally available setups.

**Table 4:** Different aperture sizes used in the numerical studies and the corresponding element pitches. The number of elements is constant (32 × 32).

Aperture Size [mm]	Element Pitch [mm]	Number of Elements
5.00	0.16	32 × 32
**9.60**	**0.30**	**32 × 32**
20.00	0.63	32 × 32
**30.08**	**0.94**	**32 × 32**
40.00	1.25	32 × 32

Bold numbers show experimentally available setups.

**Table 5:** Lateral and axial FWHM values, SNR, and localization errors for DMSAI reconstructions with 32 × 32 elements on different aperture sizes for numerical and experimental data. All simulations were performed with a frequency of 4 MHz and an opening angle corresponding to an element size of 0.88 × 0.88 mm^2^. Experimental data with 30.08 mm aperture correspond to the 4 MHz array.

Aperture Size [mm]	FWHM Lateral [mm]		FWHM Axial [mm]		SNR [dB]		Localization Error [mm]
	Sim	Exp	Sim	Exp	Sim	Exp	Sim
5.00	4.68	-	66.76	-	11.37	-	48.50
9.60	1.70	-	50.98	-	16.44	-	3.00
20.00	0.84	-	11.02	-	21.95	-	0.90
30.08	0.74	0.71	6.90	6.92	25.40	22.68	0.40
40.00	0.72	-	5.08	-	27.27	-	0.10

**Table 6:** Lateral and axial FWHM values, SNR, and localization errors for DMSAI reconstructions with 32 × 32 elements on different aperture sizes for numerical and experimental data. All simulations were performed with a frequency of 2.84 MHz and an opening angle corresponding to an element size of 0.24 × 0.24 mm^2^. Experimental data with 9.6 mm aperture correspond to the 3 MHz array.

Aperture Size [mm]	FWHM Lateral [mm]		FWHM Axial [mm]		SNR [dB]		Localization Error [mm]
	Sim	Exp	Sim	Exp	Sim	Exp	Sim
5.00	5.50	-	73.50	-	8.84	-	32.80
9.60	2.28	2.15	62.68	61.24	14.34	14.07	16.30
20.00	0.86	-	12.70	-	20.57	-	0.90
30.08	0.60	-	5.78	-	26.04	-	0.20
40.00	0.48	-	3.52	-	29.70	-	0.10

**Table 7:** Lateral and axial FWHM values as well as SNR, localization errors, and reconstruction times for DMSAI reconstructions with different numbers of elements for numerical and experimental data (obtained from the 4 MHz array, source located at (0/0/50) mm).

Number of Elements	Pitch [λ]	FWHM Lateral [mm]		FWHM Axial [mm]		SNR [dB]		Localization Error [mm]	Reconstruction Time [s]
		Sim	Exp	Sim	Exp	Sim	Exp	Sim	
63 × 63	1.27	0.74	-	6.96	-	25.28	-	0.40	50,708
32 × 32	2.54	0.74	0.71	6.90	6.92	25.40	22.68	0.40	7989
16 × 16	5.08	0.74	0.69	6.90	6.70	25.46	22.67	0.40	1913
11 × 11	7.62	0.74	0.67	6.84	6.64	25.76	21.73	0.70	805
8 × 8	10.16	0.71	0.71	6.70	6.52	25.25	20.27	0.40	413

## Data Availability

The data presented in this study are partly available on request from the corresponding author. The data are not publicly available due to privacy.

## Appendix A

**Table A1:** Lateral and axial FWHM values, SNR, and localization errors for DMSAI reconstructions with different numbers of elements for numerical and experimental data (obtained from the 4 MHz array, source located at (0.9/−5.3/50) mm).

Number of Elements	FWHM Lateral [mm]		FWHM Axial [mm]		SNR [dB]		Localization Error [mm]
	Sim	Exp	Sim	Exp	Sim	Exp	Sim
63 × 63	0.76	-	6.06	-	24.89	-	0.80
32 × 32	0.76	0.87	5.96	6.76	25.03	18.36	0.80
16 × 16	0.76	0.87	5.86	6.50	24.99	18.43	0.70
11 × 11	0.74	0.86	5.66	6.34	25.29	17.27	0.70
8 × 8	0.77	0.89	5.66	6.62	24.72	16.09	1.00

**Table A2:** Lateral and axial FWHM values, SNR, and localization errors for DMSAI reconstructions with different numbers of elements for numerical data (source located at (6/−5/50) mm).

Number of Elements	FWHM Lateral [mm]	FWHM Axial [mm]	SNR [dB]	Localization Error [mm]
	Sim	Sim	Sim	Sim
63 × 63	0.83	5.34	24.38	0.70
32 × 32	0.83	5.40	24.45	0.70
16 × 16	0.83	5.30	24.35	0.80
11 × 11	0.82	5.20	24.63	0.70
8 × 8	0.85	4.92	23.88	0.60

**Table A3:** Lateral and axial FWHM values, SNR, and localization errors for DMSAI reconstructions with different numbers of elements for numerical data (source located at (0/0/40) mm).

Number of Elements	FWHM Lateral [mm]	FWHM Axial [mm]	SNR [dB]	Localization Error [mm]
	Sim	Sim	Sim	Sim
63 × 63	0.72	5.50	26.34	0.30
32 × 32	0.72	5.42	26.27	0.20
16 × 16	0.72	5.42	26.33	0.20
11 × 11	0.72	5.18	26.55	0.60
8 × 8	0.73	5.22	25.99	0.60
